# The muscle–intervertebral disc interaction mediated by L-BAIBA modulates extracellular matrix homeostasis and PANoptosis in nucleus pulposus cells

**DOI:** 10.1038/s12276-024-01345-5

**Published:** 2024-11-07

**Authors:** Tianyu Qin, Ming Shi, Chao Zhang, Jiajun Wu, Zhengqi Huang, Xiaohe Zhang, Shuangxing Li, Yuliang Wu, Weitao Han, Bo Gao, Kang Xu, Song Jin, Wei Ye

**Affiliations:** 1 Department of Orthopedics, The Eighth Affiliated Hospital of Sun Yat-sen University, Shenzhen, 528406 China; 2 Department of Spine Surgery, Sun Yat-sen Memorial Hospital of Sun Yat-sen University, Guangzhou, 510120 China; 3 Guangdong Provincial Key Laboratory of Malignant Tumor Epigenetics and Gene Regulation, Medical Research Center, Sun Yat-sen Memorial Hospital, Sun Yat-sen University, Guangzhou, 510120 China

**Keywords:** Cell death, Transcriptomics, Mechanisms of disease

## Abstract

Upon engaging in physical activity, skeletal muscle synthesizes myokines, which not only facilitate crosstalk with various organs, including the brain, adipose tissue, bone, liver, gut, pancreas, and skin but also promote intramuscular signaling. Crosstalk is vital for maintaining various physiological processes. However, the specific interactions between skeletal muscle and intervertebral discs remain largely unexplored. β-Aminoisobutyric acid (BAIBA), an exercise-induced myokine and a metabolite of branched-chain amino acids in skeletal muscle, has emerged as a key player in this context. Our study demonstrated that exercise significantly elevates BAIBA levels in skeletal muscle, plasma, and nucleus pulposus (NP) tissues. Moreover, exercise enhances extracellular matrix (ECM) synthesis in NP tissues and upregulates L-BAIBA synthase in skeletal muscle. Both in vivo and in vitro evidence revealed that L-BAIBA impedes PANoptosis and ECM degradation in NP cells by activating the AMPK/NF-κB signaling pathway. These findings suggest that exercise, coupled with the resulting increase in L-BAIBA, may serve as an effective intervention to decelerate the progression of intervertebral disc degeneration (IDD). Consequently, L-BAIBA, which originates from skeletal muscle, is a promising new therapeutic approach for IDD.

## Exercise boosts myokine levels to combat disc degeneration

Intervertebral disc degeneration affects many people globally. Researchers investigated how exercise and a muscle-produced molecule, L-BAIBA, can slow IDD. They used human tissue samples, cell cultures, and rat models to study how exercise affects IDD and how L-BAIBA protects disc cells. The study combined lab and animal research, using human disc tissues and rats to understand disc degeneration and exercise effects. They found exercise increases L-BAIBA production, which benefits disc cells by promoting essential disc components and reducing cell death, a degeneration contributor. This could lead to non-invasive IDD treatments using the body’s exercise responses. The results showed exercise and L-BAIBA improved disc health by increasing essential disc components and reducing cell death. They suggest L-BAIBA could be a new IDD treatment, using exercise benefits at a molecular level. This summary was initially drafted using artificial intelligence, then revised and fact-checked by the author.

## Introduction

Intervertebral disc degeneration (IDD) is a primary contributor to low back pain (LBP), a condition with significant physical and mental impacts on patients. Clinically, IDD manifests with symptoms such as limb radiating pain, sensory abnormalities, and muscle strength loss, which collectively exacerbate patient distress^1^. The intervertebral disc (IVD) is composed of the nucleus pulposus (NP), fibrous annulus (AF), and cartilaginous endplates, with the physiological function of the NP being particularly critical^2^. Although the exact pathogenesis of IDD is not fully understood, research indicates that factors such as inflammatory infiltration, oxidative stress, and programmed cell death are pivotal in its progression^3–5^. These elements contribute to the reduction of extracellular matrix (ECM) components such as aggrecan (ACAN) and type II collagen (COL2A), as well as to the upregulation of ECM-degrading enzymes such as disintegrin-like and metalloprotease with thrombospondin type-1 motif enzymes (ADAMTSs) and matrix metalloproteinases (MMPs). Current clinical interventions primarily offer symptomatic relief without preventing the progression of IDD^6^. Thus, understanding the pathophysiology of IDD and identifying effective therapeutic targets remain imperative.

Exercise is heralded for its myriad health benefits, and international guidelines for LBP treatment suggest that appropriate exercise regimens can alleviate symptoms of LBP and restore impaired motor function^7^. Exercise-induced spinal loading augments fluid circulation to intervertebral discs, thereby improving nutrient metabolite exchange with the discs^8^. An increasing number of studies have advanced our understanding of the physiological mechanisms by which exercise exerts positive effects on intervertebral discs. For example, a study revealed that running enhanced lumbar IVD magnetic resonance T2 time with a stride speed of 2 m/s (akin to jogging or brisk walking), substantiating the efficacy of running in augmenting NP water hydration^9^. Furthermore, running has been shown to increase the number of NP cells and stimulate collagen and aggrecan synthesis in IVDs in rat models^10–12^. Conversely, conflicting results have surfaced from a randomized controlled study that reported a lack of favorable effects of exercise on the intervertebral disc^13^. Similarly, no significant alterations in the levels of proteoglycans or collagen in the NP were observed after 15 weeks of running in beagles^14^. Thus, the exact relationship between exercise and IDD warrants further exploration.

The health-promoting effects of exercise extend beyond physical fitness, with skeletal muscle acting as a vital secretory organ. Skeletal muscle releases myokines that not only regulate its own metabolism but also have systemic effects on various organs, including bones, the liver, and the brain, through the circulatory system^15,16^. Branched-chain amino acids (BCAAs), which are essential amino acids predominantly metabolized in skeletal muscle, constitute half of the muscle’s total amino acid intake. β-Aminoisobutyric acid (BAIBA), a small molecule derived from BCAAs, has emerged as a novel endogenous protective myokine^17^. BAIBA is instrumental in several biological processes, notably reducing inflammation, inhibiting oxidative stress, and preventing cell death^18–20^, all of which are crucial factors in the progression of IDD. Additional myokines, such as irisin, IL-6, and IGF-1, have also been implicated in IDD pathogenesis^21–23^. Nevertheless, the specific relationship between BAIBA and IDD has yet to be elucidated.

Apoptosis, pyroptosis, and necroptosis, all of which are forms of programmed cell death (PCD), are implicated in the progression of IDD^24–26^. The term “PANoptosis,” conceptualized by Kanneganti et al. in 2019, refers to the phenomenon in which cells undergo apoptosis, pyroptosis, and necroptosis simultaneously. This intricate form of cell death was initially observed in macrophages responding to the influenza A virus^27,28^. PANoptosis combines the salient characteristics of pyroptosis, apoptosis, and necroptosis but remains a distinct entity from each process in isolation. Diverse triggers ranging from infectious pathogens to shifts in the intracellular environment, such as pronounced cytokine release consequent to cell death, provoke PANoptosis^29^. For example, elevated tumor necrosis factor-alpha (TNFα) during SARS-CoV-2 infection has been shown to induce PANoptosis in bone marrow-derived macrophages^30^. Moreover, the upregulation of proinflammatory mediators such as TNFα plays a crucial role in IDD progression^31^. These findings suggest a potential association between PANoptosis and the pathogenesis of IDD.

In our study, we assessed the impact of exercise and the muscle-secreted myokine L-BAIBA on intervertebral disc degeneration. The data demonstrated that exercise mitigates the progression of IDD and that L-BAIBA significantly contributes to the promotion of extracellular matrix synthesis and the inhibition of PANoptosis in nucleus pulposus cells. These findings suggest that L-BAIBA may represent a novel therapeutic approach for the management of IDD.

## Materials and methods

### Collection of clinical specimens

The NP tissue used in this study was obtained from patients requiring internal fixation for spinal fusion at Sun Yat-sen Memorial Hospital of Sun Yat-sen University. The patients’ diseases included lumbar disc herniation, lumbar spinal stenosis, and lumbar spondylolisthesis. NP tissue from the central region of the IVD was collected during the operation, washed with saline to remove the adherent blood, immediately immersed in 4% paraformaldehyde for 48 h, dehydrated and paraffin-embedded for sectioning. The experimental design and protocol of this part of the study were reviewed and approved by the Ethics Committee of Sun Yat-sen Memorial Hospital (SYSKY-2023-956-01). In this study, magnetic resonance imaging (MRI) images of patients were obtained from an imaging system, and the IDD grade was assessed according to the Pfirrmann classification.

### Cell culture

Male Sprague‒Dawley (SD) rats were killed by intraperitoneal injection of an overdose of pentobarbital. The caudal vertebrae of the rats were cut at the Co7/Co8 intervertebral space, and the caudal skin and paravertebral musculature were carefully separated until the structure of the fibrous annulus could be clearly observed. The IVD was incised with a sharp blade, and the jelly-like nucleus pulposus was removed with forceps and placed in tubes containing 10% FBS in DMEM. After the NP tissue was centrifuged at 1000 rpm for 5 min, the medium was discarded, 0.2% protease was added, and the mixture was digested for 45 min at 37 °C. At the end of digestion, DMEM containing 10% FBS was added, the mixture was centrifuged at 1000 rpm for 5 min, and the supernatant was discarded. The tissues were digested with 2.5% type II collagenase in a 37 °C incubator for 15 min. After digestion, the NP tissues were washed with PBS. Finally, the digested NP tissue was cultured in DMEM containing 1% penicillin/streptomycin and 10% FBS at 37 °C.

### Cell viability assay

Rat NP cells were inoculated evenly into 96-well plates, and when the cells were completely attached to the dishes, different concentrations (1, 10, 50 and 100 μM) of L-BAIBA (Sigma–Aldrich, United States) and 50 ng/ml TNFα were added, and the cells were incubated for 24, 48 and 72 h. After that, the original medium was discarded, 100 µl of DMEM containing CCK-8 reagent (Hanbio, China) was added, and the cells were incubated at 37 °C for 1 h. Cell viability was calculated by measuring the absorbance at 450 nm with an optical densitometer.

### High-density culture and alcian blue staining

NP cells were digested and diluted to a density of 10,000 cells/10 μl, and then, 10 μl of cell suspension was added to the center of the 24-well plate and incubated in a 37 °C incubator for 1 h. One milliliter of DMEM containing 1% insulin transferrin selenium +2% FBS was added. Then, the cells were treated with L-BAIBA, TNFα and Compound C (Selleck, United States). After 5 days, the cells were fixed with 4% paraformaldehyde for 10 min, treated with acidification solution for 5 min, and finally stained with alcian blue (Solarbio, China).

### Animal models

The SD rats used in this study were purchased from the Animal Experiment Centre of Sun Yat-sen University, and all the animal experiments were conducted under the supervision of the Institutional Animal Care and Use Committee (IACUC) of Sun Yat-sen University and approved by the Institutional Research Ethics Committee of Sun Yat-sen University (SYSU-IACUC-2023-001583). SD rats were randomly divided into three groups: the sham surgery (sham), annulus fibrosus puncture (AFP) and AFP + L-BAIBA groups. After anesthesia, the rats were placed flat on an operating table in the prone position. AFP was performed using a 20 G needle, which was inserted into the Co7/8 intervertebral disc vertically, rotated 360° clockwise, and held for 30 s. A Hamilton syringe with a 34 G needle was used to inject 2 µl of L-BAIBA (10 µg/ml) into the IVD in the AFP + L-BAIBA group once a week for 4 weeks. In addition, IVDs were punctured with 34 G needles in the AFP and sham groups. At the end of the experiment, MRI scans of the caudal vertebrae were performed on all the rats. After MRI, the rats were euthanized by the administration of 3% sodium pentobarbital (0.4 ml/100 g), after which the plasma, intravertebral discs and gastrocnemius muscles were collected.

### Running schemes

The rats in the exercise group underwent a one-week adaptive training program, starting with 10 min of running per day, increasing by 10 min per day up to 60 min, and then started formal training: 60 min per day for 5 weeks at a speed of 16.7 m/min. The running schemes were selected with reference to previous studies^10,12^. The control rats were only allowed to move around in the cage.

### Transcriptomic analysis of rat NP cells

NP cells were divided into two groups: the TNFα group and the TNFα + L-BAIBA group. Total RNA was extracted from rat NP cells using TRIzol reagent, and the HaploX Genomics Center was commissioned to perform mRNA transcriptome sequencing. The raw data were subjected to expression difference analysis and functional enrichment analysis after data quality control and reference genome comparison. The threshold was set as follows: | log2 (fold change)| >1 and *p* < 0.05.

### Molecular docking

The X-ray crystal structures of AMPKα1 (PDB: 6C9J), AMPKα2 (PDB: 2H6D), AMPKβ1 (PDB: 6C9F), AMPKβ2 (PDB: 6B2E) and AMPKγ1 (PDB: 4RER) were retrieved from the Protein Data Bank. The predicted structures of AMPKγ2 and AMPKγ3 were generated by AlphaFold. The protonation state of L-BAIBA (PubChem ID: 439434) was set at pH = 7.4, and L-BAIBA was expanded to 3D structures using Open Babel. AutoDock Tools (ADT3) were applied to prepare and parametrize the receptor protein and ligands. The docking grid documents were generated using AutoGrid in sitemap, and AutoDock Vina (1.2.0) was used for docking simulation. The optimal pose was selected to analyze the interaction. Finally, the protein‒ligand interaction figure was generated in PyMOL.

### Statistical analysis

The data were collected from at least three independent experiments and are expressed as the means ± standard deviations. Unpaired two-tailed Student’s *t* tests were used to compare differences between two groups, whereas one-way ANOVA was used to evaluate differences between multiple groups. A value of *p* < 0.05 was considered statistically significant.

Detailed descriptions of the other materials and methods are available in the Supporting Information.

## Results

### Treadmill exercise retards AFP-induced IDD in rats

**Fig. 1:**
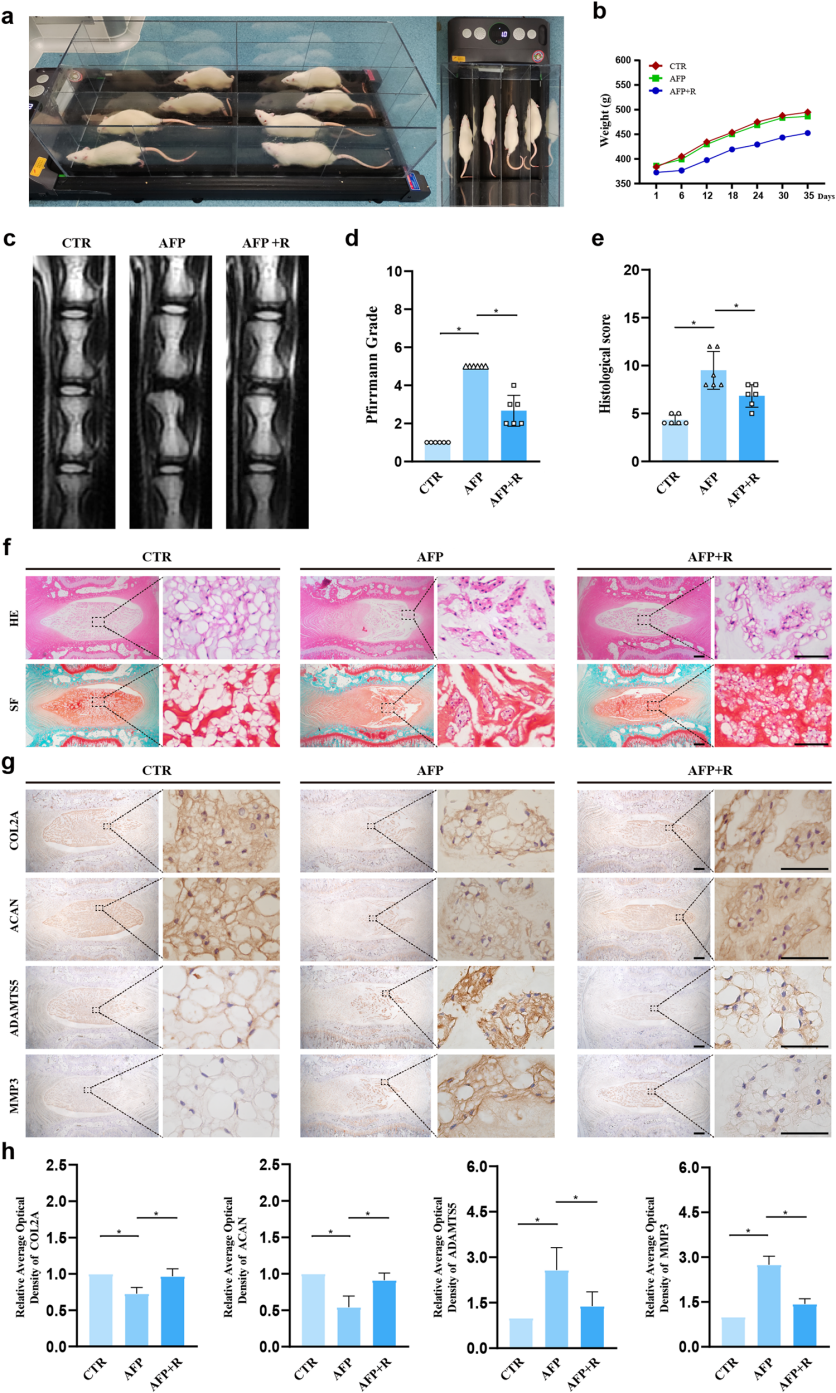
Running retards AFP-induced disc degeneration in rats. **a** Treadmill for SD rats. **b** Body weight changes in SD rats in three groups (CTR, AFP and AFP + R). **c** MRI scans of the caudal spine in SD rats. **d** Pfirrmann scores based on MRI scans of the caudal spine in SD rats. **e** Histological scoring of IDD in SD rats based on hematoxylin‒eosin (HE) staining. **f** Slices of the rat caudal vertebra were subjected to HE, safranin O and fast green (SF) staining. Scale bar: 50 μm. **g** Respective immunohistochemical (IHC) staining results for COL2A, ACAN, ADAMTS5 and MMP3 in the three groups (CTR, AFP and AFP + R). Scale bar: 20 μm. **h** Semiquantitative and statistical analysis of IHC results for COL2A, ACAN, ADAMTS5 and MMP3. The data are shown as the means ± SDs; *p < 0.05.

To elucidate the potential ameliorative effects of treadmill exercise on AFP-induced IDD, we conducted a regimen of treadmill exercise in rats, as depicted in Fig. 1a. After five weeks, we observed a trend toward reduced body weight in the AFP + Running (AFP + R) group compared with the Control (CTR) and AFP groups, as illustrated in Fig. 1b. However, the reduction did not appear to be statistically significant (CTR vs. AFP + R, *p* = 0.23; AFP vs. AFP + R, *p* = 0.32). MRI analysis demonstrated that the T2-weighted imaging signal intensity of the IVDs in the CTR group displayed a normal white high signal (Fig. 1c). In contrast, the IVDs in the AFP group presented a black low signal, whereas the IVDs in the AFP + R group presented a gray intermediate signal, which was an intermediary between those in the CTR and AFP groups. Further assessment using the MRI-based Pfirrmann grading system revealed a lower score in the AFP + R group than in the AFP group, indicating mitigated IDD severity (Fig. 1d). The histological analysis revealed that after puncturing, the disc presented a reduced number of NP cells, blurred boundaries between the NP and the AF, and inward protrusions of the AF in a serpentine pattern according to hematoxylin and eosin (HE) and safranin O and fast green (SF) staining (Fig. 1e, f). These pathological manifestations were significantly ameliorated in the AFP + R group. Additionally, IHC analysis of ECM-synthesizing enzymes (COL2A, ACAN) and catabolic enzymes (ADAMTS5, MMP3) revealed elevations of COL2A and ACAN expression levels by 24.1% and 36.9%, respectively, with concurrent reductions of 46.2% in ADAMTS5 and 47.8% in MMP3 expression in the AFP + R group compared with the AFP group, suggesting an anabolic shift in extracellular matrix homeostasis (Fig. 1g, h).

### Exercise promotes L-BAIBA production and secretion in the skeletal muscle of rats

**Fig. 2:**
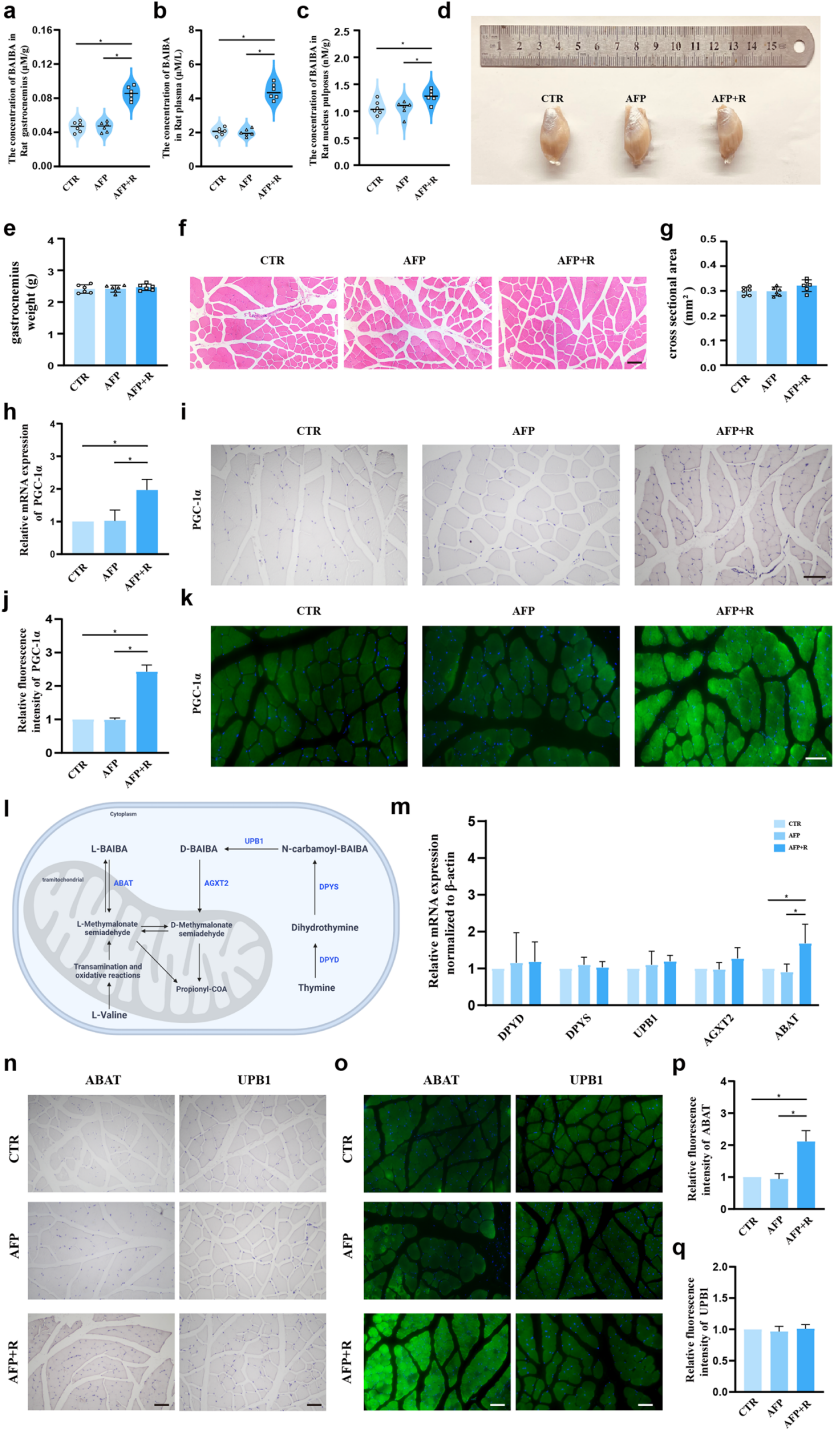
Running promotes L-BAIBA production and secretion in rats. **a**, **b**, **c** Levels of BAIBA in skeletal muscle, plasma and NP tissues of SD rats measured by liquid chromatography‒mass spectrometry (LC‒MS). **d** Representative morphological images of the gastrocnemius muscle of SD rats in the three groups (CTR, AFP and AFP + R). **e** Gastrocnemius muscle weights of SD rats in the three groups (CTR, AFP and AFP + R). **f** HE staining of the gastrocnemius muscle of SD rats in the three groups (CTR, AFP and AFP + R). Scale bar: 100 μm. **g** ANOVA was used to compare the cross-sectional areas of the gastrocnemius muscle in the three groups (CTR, AFP and AFP + R). **h** Detection of the mRNA expression levels of PGC-1α in the gastrocnemius muscle by RT‒qPCR. **i** Respective IHC images of PGC-1α in the gastrocnemius muscle. Scale bar: 100 μm. **j** Semiquantitative and statistical analyses of immunofluorescence (IF) staining for PGC-1α in (**k**). **k** Representative IF images of PGC-1α in the gastrocnemius muscle. Scale bar: 100 μm. **l** Schematic diagram of BAIBA production and metabolism. **m** RT‒qPCR detection of the mRNA expression levels of enzymes related to BAIBA synthesis (DPYD, DPYS, UPB1, AGXT2 and ABAT) in the gastrocnemius muscle. **n** IHC staining of L-BAIBA synthase ABAT and D-BAIBA synthase UPB1 in the gastrocnemius muscle. Scale bar: 100 μm. **o** Representative IF images of ABAT and UPB1 in the gastrocnemius muscle. Scale bar: 100 μm. **p**, **q** Semiquantitative and statistical analyses of IF staining for ABAT and UPB1 in (**o**). The data are shown as the means ± SDs; *p < 0.05.

Consistent with previous studies indicating that exercise and contracted skeletal muscle enhance BAIBA production and secretion^20,32^, we observed a significant increase in skeletal muscle BAIBA levels after exercise using LC‒MS to measure BAIBA levels (Fig. 2a), which was accompanied by increased plasma levels of BAIBA (CTR: 2.06 ± 0.22 μM/L; AFP: 2.00 ± 0.21 μM/L; AFP + R: 4.41 ± 0.46 μM/L, as depicted in Fig. 2b). Moreover, in contrast with the CTR and AFP groups, the AFP + R group presented modest increases in BAIBA levels in the NP tissue (Fig. 2c). As shown in Supplementary Fig. 1, BAIBA levels did not differ in human NP tissue with different degrees of degeneration. Examination of gastrocnemius muscle mass revealed no significant alterations across the groups (Fig. 2d, e), and HE staining confirmed no substantial change in muscle cross-sectional area due to exercise (Fig. 2f, g). PGC-1α is known to regulate muscle adaptation to exercise and promote BAIBA secretion^33^; therefore, we evaluated PGC-1α mRNA levels in the gastrocnemius muscle by RT‒qPCR. As expected, PGC-1α expression was upregulated following exercise (Fig. 2h), which was confirmed through IHC and IF analyses (Fig. 2i–k). To elucidate the enantiomers of BAIBA present, we measured the expression levels of enzymes associated with L-BAIBA synthesis (ABAT) and D-BAIBA synthesis (DPYD, DPYS, UPB1, AGXT2) (Fig. 2l). Notably, only the ABAT mRNA level significantly increased in the gastrocnemius muscle postexercise (Fig. 2m). The results of IHC and IF also confirmed an increase in ABAT expression in the AFP + R group, whereas UPB1 expression remained relatively unchanged across all groups (Fig. 2n–q). Thus, our results imply that the increase in L-BAIBA postexercise may contribute significantly to the retardation of IDD development.

### L-BAIBA improves ECM synthesis in rat NP cells under TNFα treatment

**Fig. 3:**
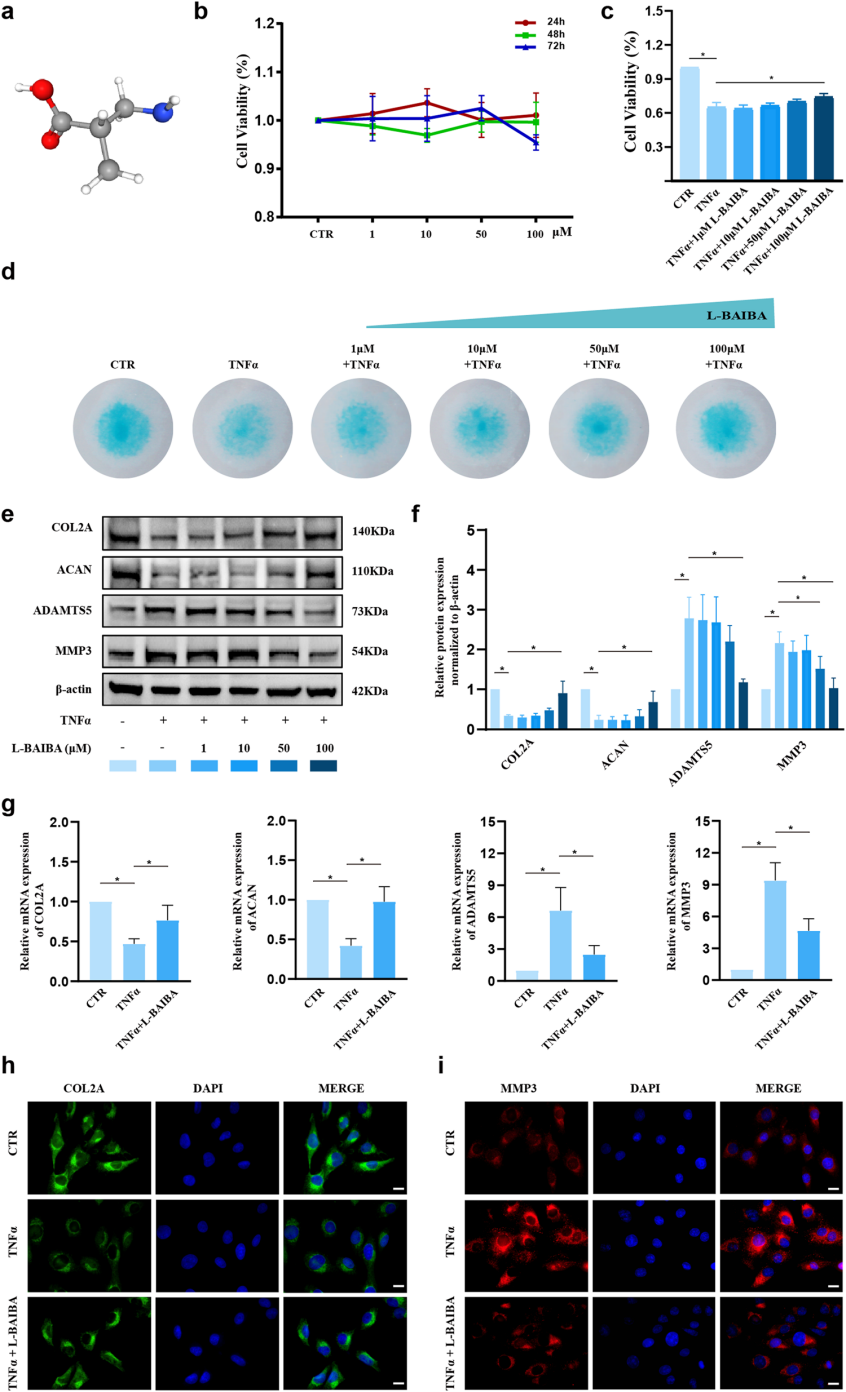
L-BAIBA restores extracellular matrix synthesis in rat NP cells under TNFα treatment. **a** Interactive chemical structure model of L-BAIBA. **b** The viability of L-BAIBA-treated rat NP cells at different concentrations and at different times was examined by CCK8 assay (*n* = 3). **c** The viability of L-BAIBA-treated rat NP cells after 72 h was detected by CCK-8 assay (*n* = 3). **d** The extracellular matrix was detected by alcian blue staining (*n* = 3). **e** The expression levels of extracellular matrix anabolic markers (COL2A and ACAN) and catabolic markers (ADAMTS5 and MMP3) were detected by Western blot (WB) in rat NP cells from each group (*n* = 4). **f** Semiquantitative and statistical analyses were performed on the Western blot in (**e**). **g** Detection of the mRNA expression levels of COL2A, ACAN, ADAMTS5 and MMP3 in the NP by RT‒qPCR (*n* = 3). **h** Representative immunofluorescence images of COL2A in rat NPs treated with TNFα or L-BAIBA (*n* = 3). Scale bar: 50 μm. **i** Representative immunofluorescence images of MMP3 in rat NPs treated with TNFα or L-BAIBA (*n* = 3). Scale bar: 50 μm. The data are shown as the means ± SDs; *p < 0.05.

To evaluate the therapeutic efficacy of L-BAIBA (Fig. 3a) in IDD, we initially performed a CCK8 assay to assess the impact of L-BAIBA on the viability of NP cells. The administration of various concentrations of L-BAIBA (0, 10, 50, or 100 μM) over different time periods (24, 48, or 72 h) did not significantly affect cell viability, as depicted in Fig. 3b. Significantly, cell viability decreased to 65% following 72 h of exposure to TNFα but subsequently improved to 74% with the addition of 100 μM L-BAIBA (Fig. 3c). Alcian blue staining, which is indicative of the degree of ECM secretion, demonstrated that L-BAIBA mitigated TNFα-induced ECM degradation, particularly at a concentration of 100 μM (Fig. 3d). Furthermore, the protein levels of COL2A and ACAN increased by 57% and 44%, respectively, compared with those in the TNFα group following treatment with 100 μM L-BAIBA. Moreover, the protein levels of ADAMTS5 and MMP3 decreased by 58% and 52%, respectively, under identical conditions (Fig. 3e, f). Therefore, a 100 μM concentration of L-BAIBA was selected for further experiments. The RT‒qPCR results further confirmed the role of L-BAIBA in increasing COL2A and ACAN mRNA expression while suppressing ADAMTS5 and MMP3 mRNA expression (Fig. 3g). Immunofluorescence assays further confirmed that L-BAIBA upregulated COL2A expression and downregulated MMP3 expression under TNFα-stimulated conditions (Fig. 3h, i).

### L-BAIBA inhibits TNFα-induced PANoptosis in rat NP cells

**Fig. 4:**
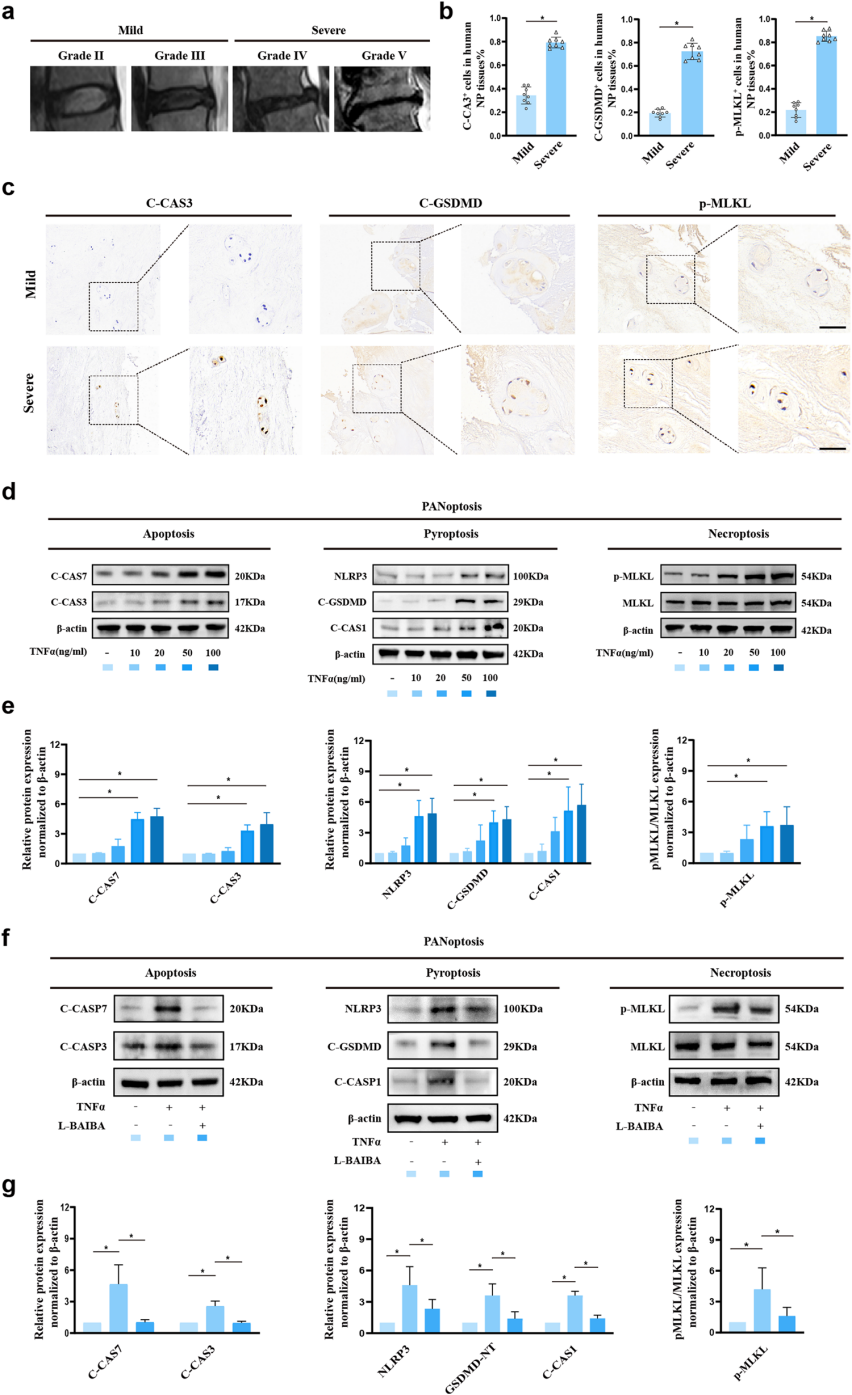
L-BAIBA inhibits TNFα-induced PANoptosis in rat NP cells. **a** Human MR images of mild and severe lumbar degeneration. **b** Ratios of C-CAS3^+^ cells, C-GSDMD^+^ cells and p-MLKL^+^ cells according to the immunohistochemical staining in (**c**). **c** Respective immunohistochemical staining of C-CAS3, C-GSDMD and p-MLKL in mildly and severely degenerated human NP tissues. Scale bar: 50 μm. **d** Expression levels of apoptosis markers (C-CAS7, C-CAS3), pyroptosis markers (NLRP3, C-GSDMD, C-CAS1) and necrosis markers (p-MLKL) in rat NP cells treated with different TNFα concentrations were detected by Western blotting (*n* = 4). **e** Semiquantitative and statistical analyses were performed on the Western blot in (**d**). **f** The expression levels of apoptosis markers, pyroptosis markers and necrosis markers in rat NP cells treated with TNFα or L-BAIBA were detected by Western blotting (*n* = 4). **g** Semiquantitative and statistical analyses were performed on the Western blot in (**f**). The data are shown as the means ± SDs; *p < 0.05.

Programmed cell death, which includes apoptosis, pyroptosis, and necroptosis, plays a critical role in reducing the number of NP cells and degrading the ECM^24^. Elevated levels of TNFα, a pivotal molecular driver of disc degeneration, exacerbate this process^31^. To investigate the correlation between PANoptosis and IDD, we first collected human NP tissues with varying degrees of degeneration (Fig. 4a). We then assessed the expression levels of apoptosis (C-CAS3), pyroptosis (C-GSDMD), and necroptosis (p-MLKL) markers by IHC. The results revealed a 45% increase in C-CAS3^+^ cells, a 53% increase in C-GSDMD^+^ cells, and a 64% increase in p-MLKL^+^ cells in NP tissues with severe degeneration compared with those with mild degeneration (Fig. 4b, c). Furthermore, WB analysis revealed that TNFα at a concentration of 50 ng/ml effectively induced PANoptosis in NP cells (Fig. 4d, e and Supplementary Fig. 2). To explore the impact of L-BAIBA on NP cell PANoptosis, we conducted WB analysis. The results demonstrated a 77% reduction in C-CAS7 and a 62% reduction in C-CAS3 protein expression in the L-BAIBA group compared with the TNFα group. For pyroptosis, the protein expression levels of NLRP3, C-GSDMD, and C-CAS1 decreased by 49%, 62%, and 61%, respectively. Additionally, L-BAIBA significantly inhibited the phosphorylation of MLKL and RIPK3, key initiators of necroptosis (Fig. 4f, g and Supplementary Fig. 3).

### L-BAIBA suppresses IDD in a rat model

**Fig. 5:**
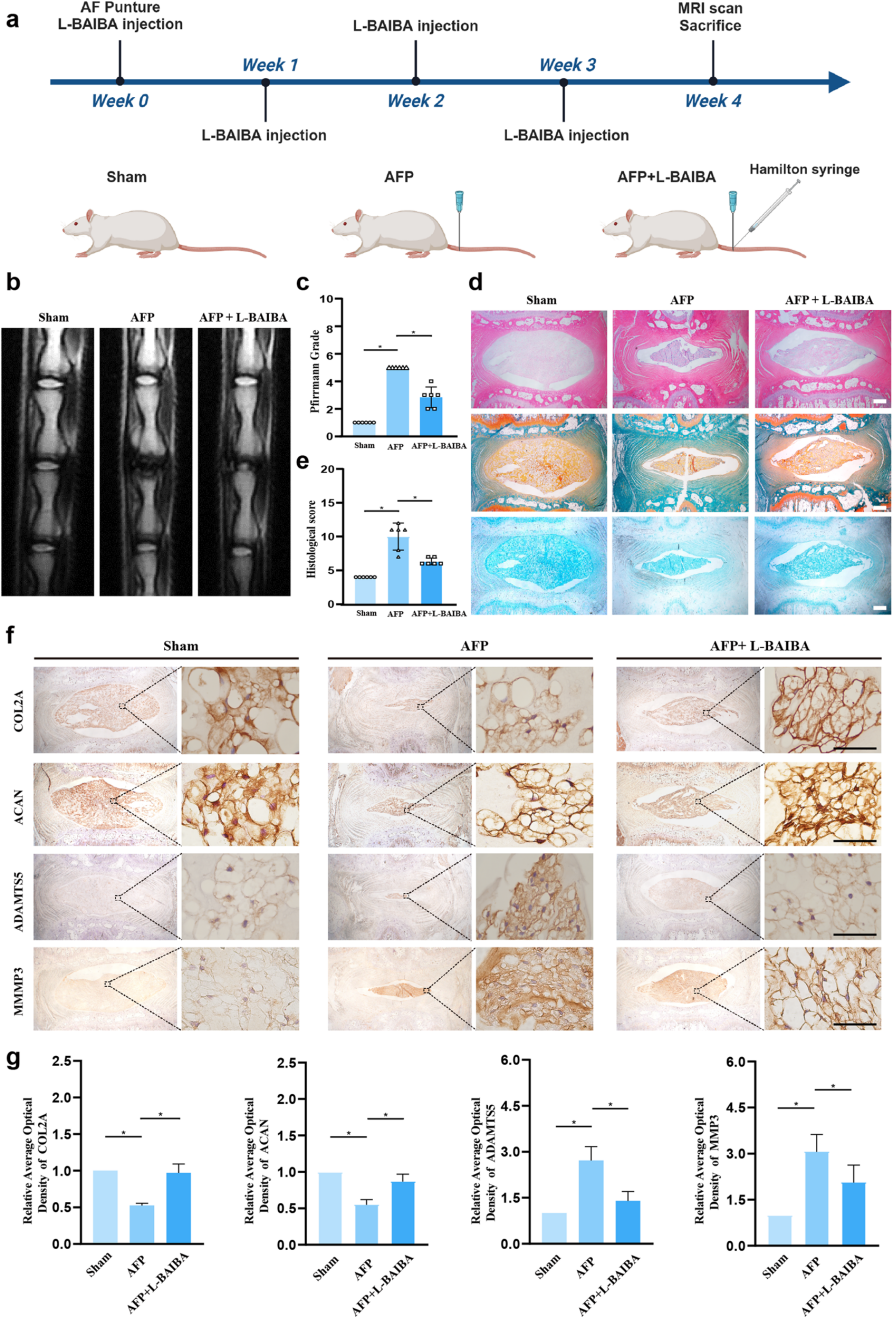
L-BAIBA alleviates metabolic disturbances in the extracellular matrix in a rat model. **a** Workflow of the SD rat experiment. **b** MRI scans of the caudal vertebra in SD rats. **c** Pfirrmann scores based on MRI scans of the caudal vertebra in SD rats. Scale bar: 50 μm. **d** Slices of the rat caudal vertebra were subjected to HE staining, SF staining and alcian blue staining. Scale bar: 500 μm. **e** Histological scoring of IDD in SD rats based on HE staining. **f** Respective immunohistochemical staining of COL2A, ACAN, ADAMTS5 and MMP3 in the three groups (CTR, AFP and AFP + L-BAIBA). Scale bar: 20 μm. **g** Semiquantitative and statistical analyses of immunohistochemical staining for COL2A, ACAN, ADAMTS5 and MMP3. The data are shown as the means ± SDs; *p < 0.05.

To further assess the protective effects of L-BAIBA in vivo, we administered 2 µl of L-BAIBA (10 µg/ml) using a Hamilton syringe. The detailed methodology and experimental groupings are depicted in Fig. 5a. MRI analysis revealed that the disc space diminished and that the signal intensity of the NP turned into a black low signal following AFP, whereas L-BAIBA partially mitigated these adverse effects (Fig. 5b). Pfirrmann score analysis revealed lower scores in the L-BAIBA group than in the AFP group, suggesting a protective role of L-BAIBA (Fig. 5c). Histological assays incorporating HE, SF, and Alcian blue staining revealed that L-BAIBA ameliorated the AFP-induced reductions in NP cell number and ECM (Fig. 5d). As shown in Fig. 5e, the histological score in the L-BAIBA group was 3.7 points lower than that in the AFP group. We also analyzed the protein expression levels of ECM synthesis markers (COL2A and ACAN) and degradation markers (ADAMTS5 and MMP3) through IHC. Compared with those in the CTR group, the expression levels of COL2A and ACAN decreased in the AFP group but were restored by L-BAIBA treatment. Conversely, the expression levels of ADAMTS5 and MMP3 increased in the AFP group but decreased in the L-BAIBA group (Fig. 5f, g).

**Fig. 6:**
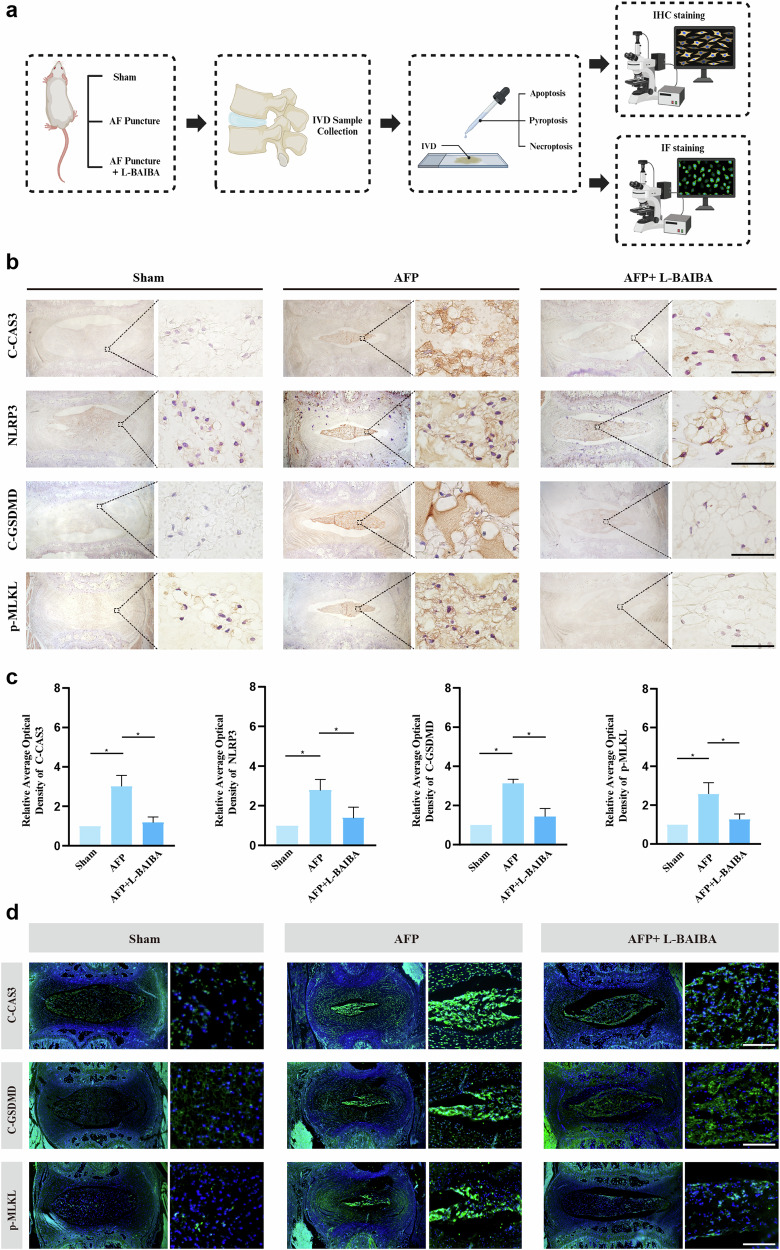
L-BAIBA suppresses PANoptosis in a rat model. **a** Schematic diagram for detecting PANoptosis in SD rats. **b** Respective immunohistochemical staining of apoptosis markers (C-CAS3), pyroptosis markers (NLRP3, C-GSDMD) and necrosis markers (p-MLKL) in the three groups (CTR, AFP and AFP + L-BAIBA). Scale bar: 20 μm. **c** Semiquantitative and statistical analyses of immunohistochemical staining for apoptosis markers (C-CAS3), pyroptosis markers (NLRP3, C-GSDMD) and necrosis markers (p-MLKL). **d** Representative immunofluorescence images of apoptosis markers (C-CAS3), pyroptosis markers (C-GSDMD) and necrosis markers (p-MLKL). Scale bar: 200 μm. The data are shown as the means ± SDs; *p < 0.05.

To explore the relationship between L-BAIBA and PANoptosis in this model, we assessed PANoptosis-relevant biomarker expression by IHC and IF (Fig. 6a). IHC analysis revealed that AFP increased the expression levels of apoptosis, pyroptosis, and necroptosis markers, which were significantly reduced following L-BAIBA treatment (Fig. 6b, c). Immunofluorescence analysis further confirmed that the expression levels of C-CAS3, C-GSDMD, and p-MLKL significantly decreased after L-BAIBA treatment (Fig. 6d).

### AMPKα1 phosphorylation is increased by L-BAIBA

**Fig. 7:**
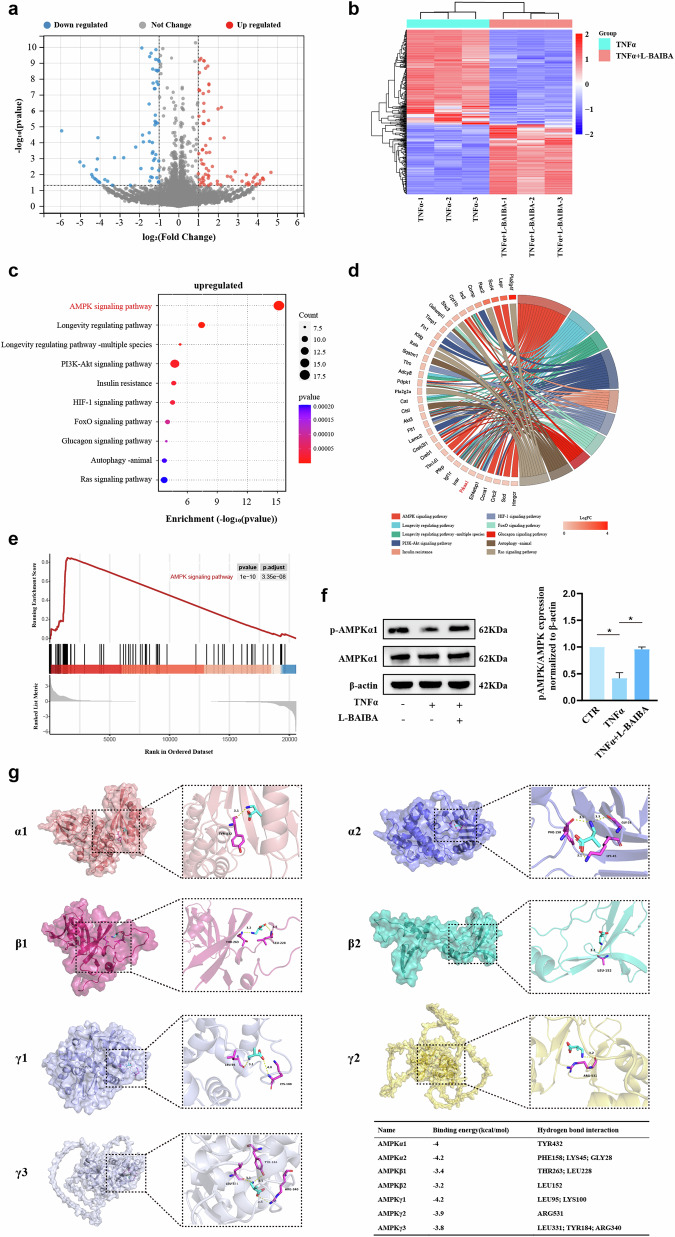
L-BAIBA promotes AMPKα phosphorylation. **a** Volcano plot of transcriptome sequencing of rat NP cells in the two groups (TNFα and TNFα + L-BAIBA). **b** Heatmap of differentially expressed genes ( | fold change | > 2, *p* < 0.05) between the TNFα and TNFα + L-BAIBA groups. **c** Dot plot for KEGG enrichment analysis based on upregulated genes (fold change > 2, *p* < 0.05) between the TNFα and TNFα + L-BAIBA groups. **d** Pie plot for KEGG enrichment analysis of upregulated genes (fold change > 2, *p* < 0.05) between the TNFα and TNFα + L-BAIBA groups. **e** GSEA of all genes in the two groups (TNFα and TNFα + L-BAIBA). **f** W**e**stern blot analysis of the protein expression of p-AMPKα/AMPKα in rat NP cells treated with TNFα or L-BAIBA. **g** Molecular docking between L-BAIBA and AMPKα. The data are shown as the means ± SDs; *p < 0.05.

To elucidate the mechanism by which L-BAIBA regulates ECM metabolism and PANoptosis in NP cells, we conducted transcriptome sequencing under treatment with 100 μM L-BAIBA and 50 ng/ml TNFα. Differential gene expression was analyzed with thresholds of |fold change | > 2 and *p* < 0.05, resulting in the identification of 211 upregulated and 155 downregulated genes (Fig. 7a, b). KEGG pathway enrichment analysis of the upregulated genes highlighted the AMPK pathway, with AMPKα1 as a pivotal constituent (Fig. 7c, d). Gene set enrichment analysis (GSEA) confirmed L-BAIBA-induced AMPK pathway activation (Fig. 7e). To validate these findings, we performed WB to assess the phosphorylation level of AMPKα1 following L-BAIBA treatment. TNFα markedly reduced the p-AMPKα1/AMPKα1 ratio, an effect that was reversed by L-BAIBA (Fig. 7f). Molecular docking assays were performed to investigate the interaction between L-BAIBA and AMPK. The three- and two-dimensional structures of the molecular docking data are presented in Fig. 7g and Supplementary Fig. 4. The docking results indicated that L-BAIBA formed hydrogen bonds with various amino acid residues across different AMPK subunits (AMPKα1, AMPKα2, AMPKβ1, AMPKβ2, AMPKγ1, AMPKγ2, and AMPKγ3). Notably, L-BAIBA was found to interact with the TYR residue of AMPKα1. Among the complexes, those formed by L-BAIBA with AMPKα1, AMPKα2, and AMPKγ1 were the most stable, exhibiting binding energies of −4 kcal/mol, −4.2 kcal/mol, and −4.2 kcal/mol, respectively.

### By activating AMPK, L-BAIBA suppresses the NF-κB signaling pathway

**Fig. 8:**
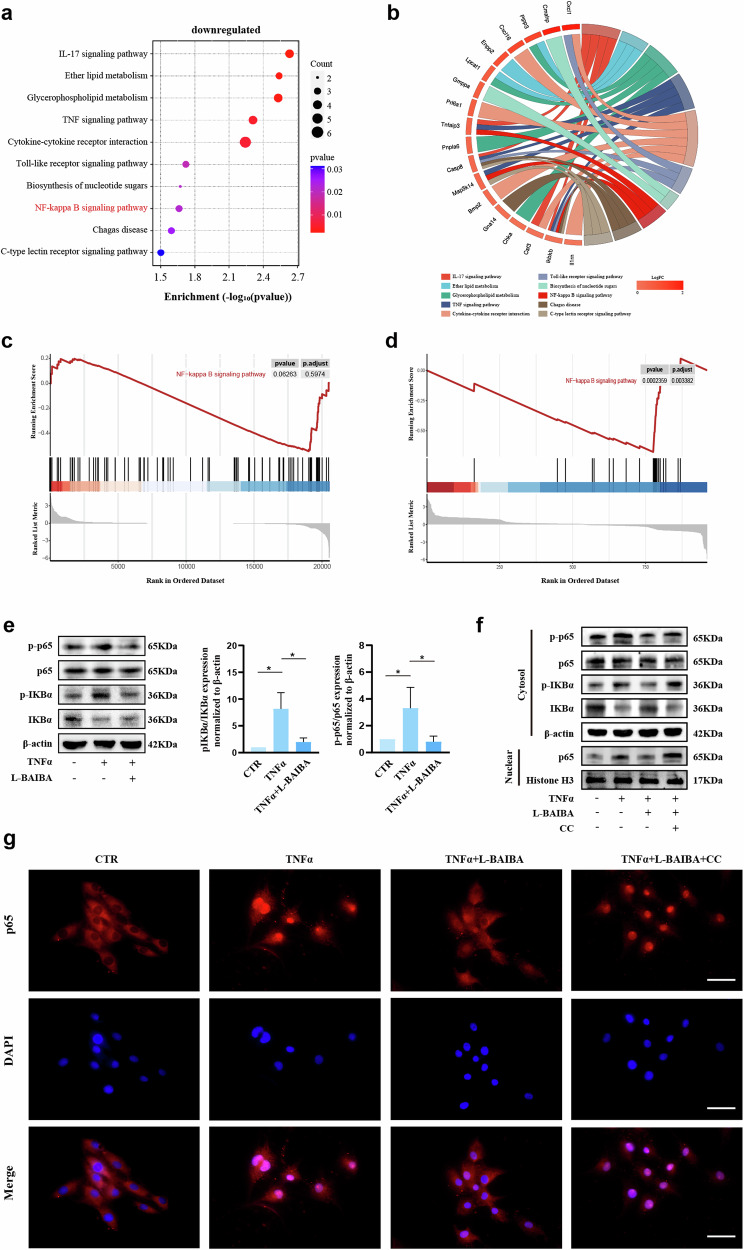
By activating AMPK, L-BAIBA suppresses the NF-κB signaling pathway. **a** Dot plot for KEGG enrichment analysis of downregulated genes (fold change < −2, *p* < 0.05) between the TNFα and TNFα + L-BAIBA groups. **b** Pie plot for KEGG enrichment analysis of downregulated genes (fold change < −2, *p* < 0.05) between the TNFα and TNFα + L-BAIBA groups. **c** GSEA of all genes in the two groups (TNFα and TNFα + L-BAIBA). **d** GSEA of all DEGs (*p* < 0.05) between the TNFα and TNFα + L-BAIBA groups. **e** Western blot analysis of the protein expression of p-p65/p65 and p-IkBα/IkBα in rat NP cells treated with TNFα or L-BAIBA. **f** Representative Western blot analysis of NF-κB activation in rat NP cells. **g** Representative images of p65 immunofluorescence in the four groups (CTR, TNFα, TNFα + L-BAIBA and TNFα + L-BAIBA + CC). Scale bar: 50 μm. The data are shown as the means ± SDs; *p < 0.05.

KEGG functional enrichment of transcriptionally sequenced downregulated genes (fold change < −2, *p* < 0.05) revealed considerable aggregation within the NF-κB signaling pathway (Fig. 8a, b). Additionally, GSEA was performed on the gene set related to the NF-κB signaling pathway. GSEA of all genes in the TNFα and TNFα + L-BAIBA groups revealed enrichment of the NF-κB pathway, although the difference did not reach statistical significance (*p* = 0.06). In contrast, GSEA of differentially expressed genes (*p* < 0.05) between the same groups revealed significant enrichment in the NF-κB pathway (*p* = 0.0002) (Fig. 8c, d). As shown in Fig. 8e, L-BAIBA decreased the TNFa-induced p-p65/p65 and p-IKBα/IKBα ratios, suggesting that L-BAIBA inhibits the activation of the NF-κB signaling pathway. Furthermore, previous studies have indicated that AMPK activation inhibits NF-κB signaling^34^. To investigate this phenomenon in NP cells, we used WB to examine NF-κB pathway markers. We observed that the AMPK inhibitor Compound C (CC) not only increased the phosphorylation of P65 and IKBα but also increased the nuclear expression of p65 (Fig. 8f). Immunofluorescence assays further confirmed that L-BAIBA delayed the TNFa-induced translocation of P65 into the nuclei of NP cells (Fig. 8g).

### L-BAIBA suppresses TNFα-induced ECM degradation, apoptosis and pyroptosis by activating AMPKα1

**Fig. 9:**
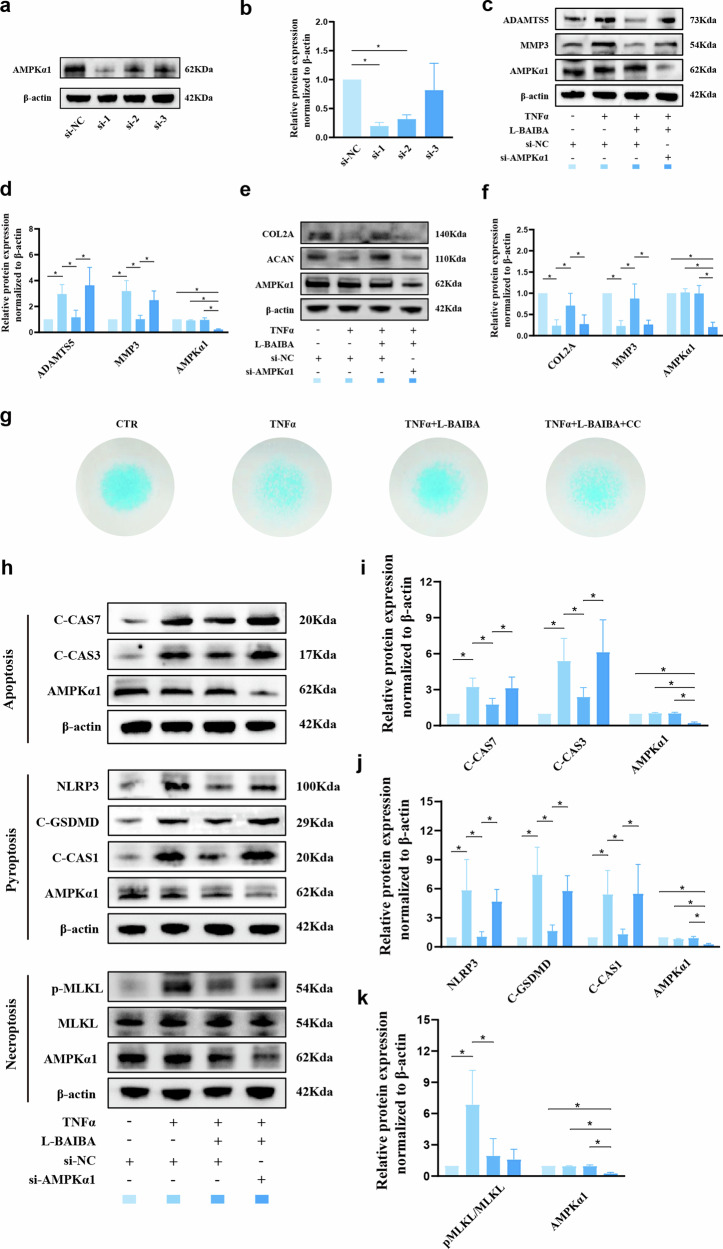
L-BAIBA suppresses TNFα-induced extracellular matrix degradation, apoptosis and pyroptosis by activating AMPKα. **a** The expression levels of AMPKα1 in rat NP cells were detected by Western blotting. **b** Western blot analysis of AMPKα1 knockdown efficiency (*n* = 3). **c** The expression levels of extracellular matrix catabolic markers (ADAMTS5 and MMP3) were detected by Western blot in rat NP cells (*n* = 4). **d** Semiquantitative and statistical analyses were performed on the Western blot in (**c**). **e** The expression levels of extracellular matrix catabolic markers (ADAMTS5 and MMP3) were detected by Western blot in rat NP cells (*n* = 4). **f** Semiquantitative and statistical analyses were performed on the Western blot in (e). **g** The extracellular matrix in each group was detected by alcian blue staining (*n* = 3). **h** The expression levels of apoptosis markers (C-CAS7 and C-CAS3), pyroptosis markers (NLRP3, C-GSDMD, and C-CAS1) and necrosis markers (p-MLKL) in rat NP cells were detected by Western blotting (*n* = 4). **i**–**k** Semiquantitative and statistical analyses were performed on the Western blot in (**h**). The data are shown as the means ± SDs; *p < 0.05.

To determine whether L-BAIBA regulates ECM metabolism and PANoptosis through AMPKα1, we transfected NP cells with siRNA to suppress AMPKα1 transcription. The silencing efficiency was evaluated by WB, which revealed the maximal efficiency of siRNA-1 (Fig. 9a, b); therefore, siRNA-1 was selected for subsequent experiments. WB analysis revealed that AMPKα1 suppression counteracted the L-BAIBA-induced reduction in ADAMTS5 and MMP3 expression (Fig. 9c, d) and reversed the L-BAIBA-induced upregulation of COL2A and ACAN (Fig. 9e, f). Alcian blue staining was then employed to evaluate ECM synthesis, which revealed that L-BAIBA-enhanced ECM synthesis was diminished by CC (Fig. 9g). Moreover, we investigated the expression of PANoptosis markers following AMPKα1 knockdown. The TNFa-induced increases in C-CAS3 and C-CAS7 expression were mitigated by L-BAIBA and then restored by si-AMPKα1 (Fig. 9h, i). Similar trends were observed for NLRP3, C-GSDMD, and C-CAS1 (Fig. 9h, j). Notably, the ratios of p-MLKL/MLKL and p-RIPK3/RIPK3, which were initially inhibited by L-BAIBA, were unaffected by AMPKα1 downregulation, suggesting that the influence of L-BAIBA on necroptosis in NP cells was independent of AMPKα1 (Fig. 9h, k and Supplementary Fig. 5).

## Discussion

Numerous studies have highlighted the beneficial impacts of physical exercise on intervertebral discs^35,36^. Nonetheless, it warrants emphasis that not every exercise modality confers equivalent benefits. High-impact athletics such as gymnastics, wrestling, and rugby can lead to lumbar disc injuries, whereas weightlifting has been associated with reduced proteoglycan production in IVDs^37^. Interestingly, swimmers and baseball participants present a greater prevalence of IDD than do runners or nonathletes, potentially because of the recurring torsional forces endemic to these sports^38^. Conversely, running is generally considered beneficial or at least nondetrimental to IVDs^39,40^. In our study, we developed a running model for SD rats involving 16.7 m/min for 60 min daily. Following a five-week regimen of consistent running, we observed a protective effect against AFP-induced IDD. This protection was evidenced by an increase in the number of NP cells, increased extracellular matrix synthesis, and reduced expression of ECM-degrading enzymes, aligning with findings from previous studies^10,11^.

Myokines, which are amino acids or peptides secreted by skeletal muscle, have garnered significant research interest. Hundreds of myokines have been identified, with a growing focus on their roles in muscle interactions with other organs, including adipose tissue, bone, liver, intestine, pancreas, blood vessels, brain, and skin^41^. Irisin, a notable myokine, inhibits NP cell senescence and promotes the expression of ECM anabolic genes while reducing ECM catabolic gene expression^21,42^. A meta-analysis revealed elevated serum levels of IL-6, another myokine, in degenerated IVD tissues compared with normal tissues, with subsequent studies elucidating the regulatory mechanisms of IL-6 in IDD^22,43^. Additionally, Deng et al. demonstrated that the overexpression of the myokine IGF-1 in rabbit intervertebral discs increased ACAN and COL2A mRNA expression^44^. These studies suggest the potential for a myokine-mediated interplay between skeletal muscle and intervertebral disc tissues. In the present study, we observed a significant increase in BAIBA levels in skeletal muscle and plasma after a five-week running regimen in SD rats, reinforcing the idea that exercise stimulates BAIBA synthesis and secretion^20,45^. Concomitantly, an increase in BAIBA levels was observed within IVDs, bolstering the notion of a musculoskeletal-disc axis. Given that small molecules such as glucose (180.16 g/mol) can diffuse into IVDs to nourish NP cells^8^, L-BAIBA, with its lower molecular weight (103.12 g/mol), could similarly diffuse into NP cells. This supposition was validated by liquid chromatography‒mass spectrometry, confirming increased BAIBA concentrations within the NP tissues postexercise in SD rats. Pertinently, the identification of the amino acid transporter proteins SLC1A5 and SLC38A2 in human NP cells suggests a potential pathway for BAIBA cellular import^46^.

Aerobic exercise is known to increase PGC-1α expression in skeletal muscle^47^. Correspondingly, our results revealed a significant increase in PGC-1α expression in skeletal muscle postexercise in rats. Notably, in vitro cultures demonstrated that myocytes overexpressing PGC-1α presented a marked increase in BAIBA in the medium. Parallel findings were observed in vivo, in which plasma BAIBA concentrations were 11-fold higher in PGC-1α transgenic mice than in PGC-1α knockout mice^33^. The enhancements in mitochondrial function associated with aerobic activity, including increases in mitochondrial density and the number and activity of mitochondrial enzymes, are closely linked to BAIBA anabolism^48,49^. Our research thus focused on the enzymatic pathways facilitating L-BAIBA and D-BAIBA production, specifically ABAT (an enzyme related to L-BAIBA synthesis) and AGXT2, UPB1, DPYS, and DPYD (enzymes related to D-BAIBA synthesis). Postaerobic conditioning, we observed a significant increase in ABAT expression in the gastrocnemius muscle of SD rats. This finding aligns with Kitase et al.‘s discovery that muscle contraction enhances the synthesis of L-BAIBA but not D-BAIBA^32^.

Adenylate-activated protein kinase (AMPK) is a heterotrimer composed of a catalytic α subunit (α1 and α2) and two regulatory subunits, β (β1 and β2) and γ (γ1, γ2, and γ3). AMPK plays crucial roles in various cellular processes, including cell growth, senescence, apoptosis, autophagy, and mitochondrial biosynthesis, by phosphorylating downstream substrates^50^. Several studies have implicated AMPK in the pathogenesis of IDD^51–53^. Furthermore, BAIBA has been shown to activate AMPK^19,54^. Our transcriptome sequencing and Western blot analysis results indicated that L-BAIBA promotes the phosphorylation of AMPKa1. Moreover, molecular docking revealed that L-BAIBA has a strong binding affinity for the α-subunit of AMPK, suggesting that L-BAIBA may regulate intervertebral disc degeneration through the activation of AMPK. Previous studies have shown that AMPK suppresses NF-κB-mediated inflammatory responses^34,55^. In our study, AMPK inhibited the nuclear translocation of p65 in NP cells, suggesting that the AMPK/NF-κB axis plays a significant role in the regulatory effect of L-BAIBA on IDD.

PANoptosis, a form of inflammatory programmed cell death, is characterized by pyroptosis, apoptosis, and necroptosis. Previous studies have shown that TNFα can induce apoptosis, pyroptosis, or necroptosis in NP cells^56–58^. In our study, we detected concurrent increases in biomarkers indicative of all three programmed cell death modalities in TNFα-challenged NP cells. Furthermore, KEGG analysis of our transcriptomic data revealed the downregulation of the TNFa pathway by L-BAIBA, suggesting a suppressive influence on PANoptosis. The NF-κB pathway, which is activated by inflammatory factors, has a well-established association with apoptosis and pyroptosis in NP cells^59,60^. Correspondingly, our data revealed that AMPK was responsive to the inhibitory effects of L-BAIBA on apoptosis and pyroptosis but not necroptosis, indicating that L-BAIBA suppresses pyroptosis and apoptosis in NP cells through the AMPK/NF-κB axis.

**Fig. 10:**
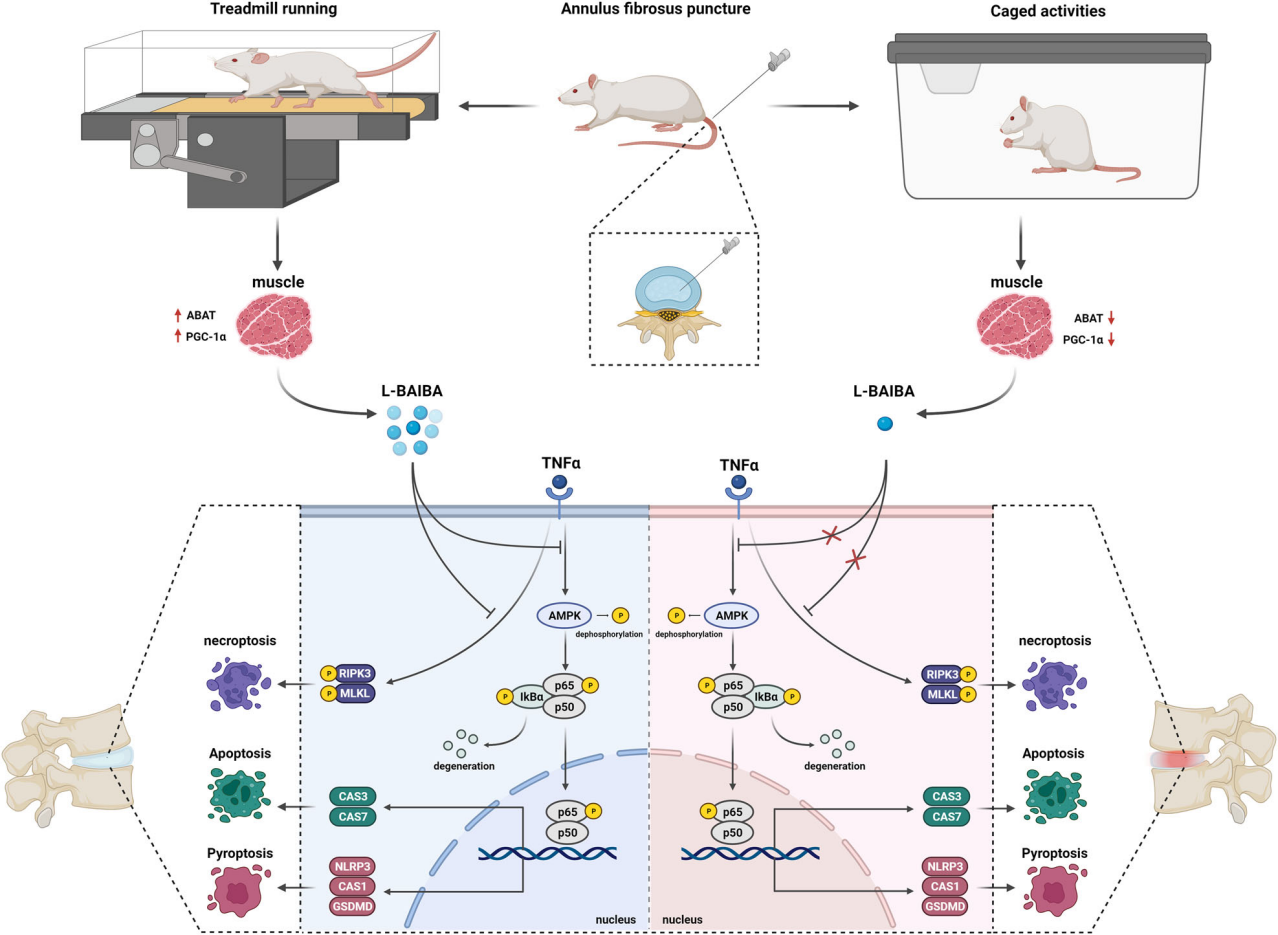
The muscle–intervertebral disc interaction mediated by L-BAIBA modulates extracellular matrix homeostasis and PANoptosis in nucleus pulposus cells.

In conclusion, our research revealed that exercise stimulates the expression of ABAT and PGC-1α within skeletal muscle, subsequently increasing the synthesis and secretion of L-BAIBA. L-BAIBA, in turn, exerts a pivotal inhibitory effect on PANoptosis and promotes extracellular matrix synthesis, predominantly via the activation of the AMPK/NF-κB axis (as depicted in Fig. 10). Based on these significant results, L-BAIBA is a promising therapeutic agent for the management of intervertebral disc degeneration.

## Supplementary information

SUPPLEMENTAL MATERIAL

## Data Availability

The data presented in the study are deposited in the Sequence Read Archive (SRA) repository, accession number PRJNA1018342.
