# Supplementary material for: The muscle–intervertebral disc interaction mediated by L-BAIBA modulates extracellular matrix homeostasis and PANoptosis in nucleus pulposus cells
Source: Exp Mol Med. 2024 Nov 7;56(11):2503–18. doi: 10.1038/s12276-024-01345-5 (PMC11612187; doi:10.1038/s12276-024-01345-5)
Supplement: Supplementary file 1 — SUPPLEMENTAL MATERIAL [file 12276_2024_1345_MOESM1_ESM.pdf]

Supplementary Information

1.Materials and methods

1.1 qRT–PCR

Cells were homogeneously seeded in 6-well plates and given corresponding stimuli, and then RNA was extracted using EZ-press RNA Purification Kit (EZBioscience, USA). RNA reverse transcription was performed by configuring 10μl of reverse transcription reaction solution according to the instructions of HiScript II Q RT SuperMix Reagent (vazyme, China). qPCR was performed using General Purpose High Sensitivity Dye-based Quantitative PCR Assay Kit (vazyme, China). The primer sequences used in this study are shown in the Supplementary Table1.

1.2 siRNA transfection

Transfection was performed when the cell density reached 50-70%. Add 125ul of DEPC water for 1OD siRNA, and the concentration of siRNA was 20μM after dissolution. siRNA and lipo3000 (ThermoFisher, USA) were diluted with Opti-MEM respectively, and the mixture formed by siRNA and lipo3000 was added into the medium, and the transfected cells were continued to be cultured for 48h-72h.Finally, the transfection efficiency was detected by WB or qPCR. The siRNA sequences were designed by IGEBIO (IGEBIO, China) (Supplementary Table 2).

1.3 Western blot analysis

Prefabricated Gel (ACE, China) was fixed in the electrophoresis tank and protein sample was added to each well. The electrophoresis was carried out at a constant voltage of 150V. The electrotransfer was carried out at a constant current of 230 mA for 2 h. The PVDF membrane was blocked with 5% BSA for 1 h. The PVDF membrane was cut according to the molecular weight of the target protein, and the cut strips were placed in the solution containing the corresponding primary antibody and incubated overnight at 4°C. The strips were washed three times in TBST solution. Goat anti-rabbit

secondary antibody at a concentration of 1:8000 was added and incubated at room temperature (RT) for 1 hour. At the end of the incubation, the strips were washed three times in TBST solution. ECL luminescent solution was added to the protein side of the strip and photographed in an imaging system. The antibodies for western blotting are shown in the Supplementary Table 3.

1.4 Histology and immunohistochemistry

Tissue samples after fixation and decalcification were entrusted to Servicebio (Wuhan, China) for paraffin embedding and sectioning. The paraffin slides were baked in an oven at 60°C for 2 hours and were immersed in xylene solution for dewaxing. The slides were then hydrated in alcohol. Pepsin was added to the tissue and incubated for 20 min at 37°. Endogenous peroxidase blocker was added and incubated for 20 minutes at RT. Tissue was blocked with 10% goat serum for 1h at RT and incubated with primary antibody (Supplementary Table 4) overnight at 4°C. On the next day, the tissues were stained with DAB after secondary antibody incubation. The slides were photographed using a microscope and at least five fields of view were taken for each slide. For HE staining, SF staining and Alcian staining, slides were dewaxed and hydrated and then stained according to the corresponding manufacturer's kit instructions.

1.5 Immunofluorescence

Rat NP cells were seeded in 24-well plates, and the cells were given corresponding stimulation. The medium was discarded, 4% paraformaldehyde was added and fixed for 30 minutes, then 0.5% Triton-100 was added for permeabilization. After the cells were blocked with 10% goat serum, the primary antibody (Supplementary Table 5) was added and incubated at 4°C overnight. On the next day, fluorescent secondary antibody (ZSGB-BIO, China) was added and incubated for 1 hour. Finally, the cells were incubated with DAPI for 5min. For tissue slides, after dewaxing and hydration, goat serum sealing, primary antibody incubation, secondary antibody incubation, DAPI incubation were performed sequentially, and finally photographs were taken using a fluorescence microscope.

1.6 Measurement of L-BAIBA levels

For plasma samples: Take 10μL of sample and add 10μL of ultrapure water, 5μL of internal standard (25μmol/L), and 40μL of isopropanol (0.1% formic acid). Mix for 5 minutes and centrifuge 10μL of supernatant at low temperature. Add 70μL of buffer salt (borax: boric acid = 8:2) and mix for 0.5 min. Add 20μL of AQC derivatiser (6 mg/mL), shake immediately for 1 min, transfer to a 55℃ oscillator for derivatisation for 10 min, and then add 400μL of water and shake for 10 min. Take 100μL of supernatant for LC-MS/MS analysis.

For skeletal muscle and NP tissue: Samples were homogenised by adding ultrapure water. Shake the extract for 1h, put it in a low temperature centrifuge, centrifuge at 4℃, 12000rpm for 10min, take 10μL of the supernatant, put it in a 1.5mL centrifuge tube, add 10μL of ultrapure water, 5μL of internal standard and 40μL of isopropanol (0.1% formic acid), and then vortex for 2 min. Place the tube in a low-temperature centrifuge and centrifuge for 10 min at 4°C and 12000 rpm. Take 10μL of the supernatant, put it in a 1.5 mL centrifuge tube, add 70μL of borate buffer salt, 20μL of AccQ Tag derivatisation reagent (Kairos Amino Acid Kit, USA), and immediately shake for 10 s. After 1 min, the derivatisation reaction was completed by hydrolysis of the excess derivatisation agent; the centrifuge tube was placed in a centrifuge tube and heated at 55°C for 10 min; and then, the reaction was diluted by adding 400μL of water. Take 100μL of supernatant for LC-MS/MS analysis.

2.Supplementary figures

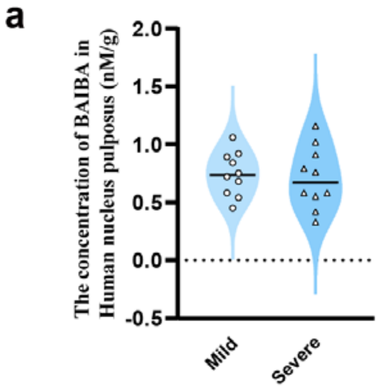

Supplementary Fig. 1 (a) Levels of BAIBA in human nucleus pulposus Tissue of Mild Degeneration and Severe Degeneration measured by Liquid Chromatograph Mass Spectrometer (LC-MC).

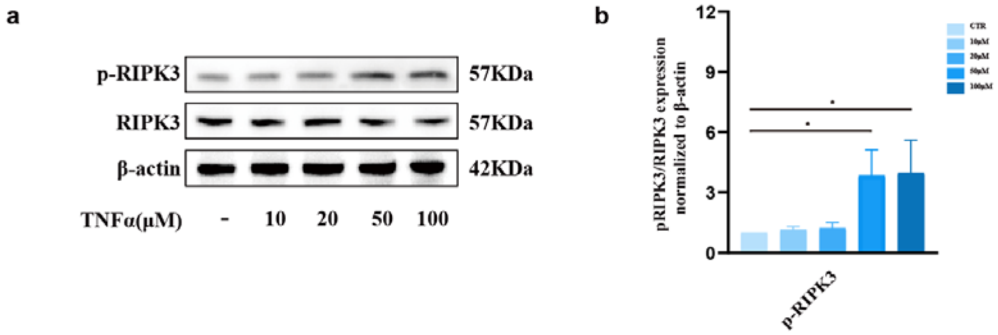

Supplementary Fig. 2 (a) Protein expression of p-RIPK3 and RIPK3 in rat NP cells treated with TNFα were detected by Western blot. (b) Semi-quantitative and statistical analyses were performed on the Western blot in (a)

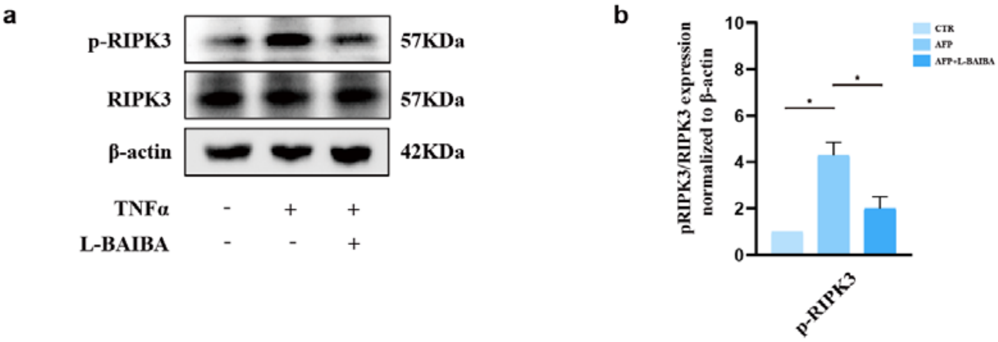

Supplementary Fig. 3 (a) Protein expression of p-RIPK3 and RIPK3 in rat NP cells treated with TNFα or L-BAIBA were detected by Western blot. (b) Semi-quantitative

and statistical analyses were performed on the Western blot in (a)

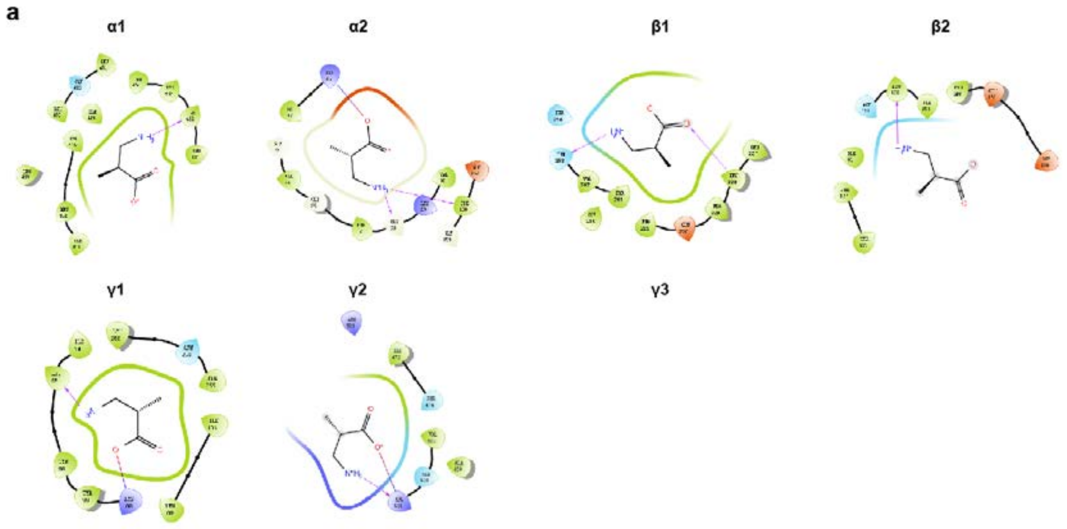

Supplementary Fig. 4 (a) Two-dimensional structure of molecular docking between L-BAIBA and AMPK (AMPKα1, AMPKα2, AMPKβ1, AMPKβ2, AMPKγ1, AMPKγ2, AMPKγ3)

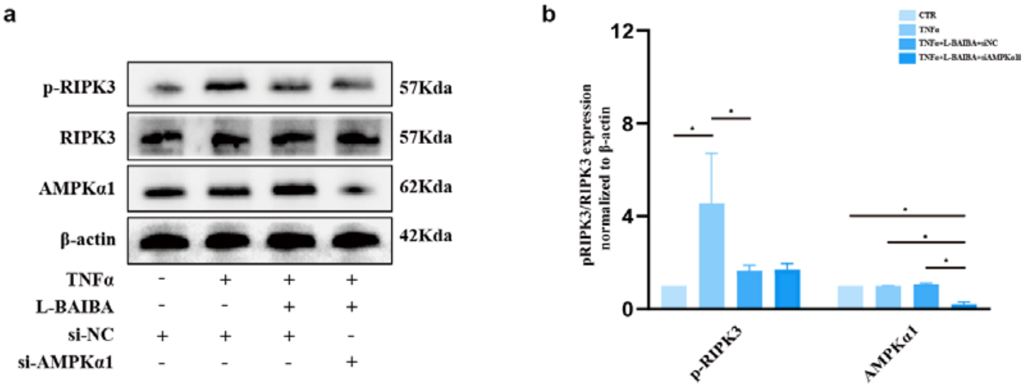

Supplementary Fig. 5 (a) Protein expression of p-RIPK3 and RIPK3 in rat NP cells were detected by Western blot. (b) Semi-quantitative and statistical analyses were performed on the Western blot in (a)

3.Supplementary table

Supplementary Table 1 Primers Used in the Present Study

Gene	Sense	Anti-sense
β-actin	TCTCTGCTCCTCCCTGTTC	ACACCGACCTTCACCATCT
ABAT	CCGCATGTTGGACCTGTATTCT	AGGGCAGGTCTGTTGATGAAAGT
AGXT2	GCAGCAGTTGTGACCACTCCAG	ACCTCAAGCACAGCAGATCCAATG
UPB1	CAGGAGACCGGCTTCTTGATT	CAGGGTCCCACAGAAATTCAAC
DPYS	GTGCACAGTGGCAAAATGGAT	GTCCCAGATCACAATGTCAGCAT
DPYD	GGTGGGCAGTGGAAAGAGG	GGCGGTCACAGCTCTCAAAG
PGC-1α	TGCTAGCGGTCCTCACAGAGA	AGGACTGGCCTCGTTGTCAGT
COL2A	CACGCTCAAGTCGCTGAA	AGCCCTGGTTGGGATCA
ACAN	ACACGGCTCCACTTGATTCTT	CTTGGTCTTTGTGACTCTTGCG
ADAMTS5	AAAACTGGCGAGTACCTT	TCCTTTGTGGCTGAATAG
MMP3	CGGTTCCGCCTGTCTCAAG	CGCCAAAAGTGCCTGTCTT

Supplementary Table 2 Sequence of si-NC and si-AMPKα1 Used in the Present Study

Sequence	Sense	Anti-sense
si-AMPKα1	CGAGUUGACUGGACAUAAAdTdT	UUUAUGUCCAGUCAACUCGdTdT
si-NC	UUCUCCGAACGUGUCACGUdTdT	ACGUGACACGUUCGGAGAAdTdT

Supplementary Table 3 The primary antibodies for western blotting

Name	Dilution	Lot Number	Company	Country	RRID
COL2A1	1:500	YT1022	Immunoway	USA	AB_2935872
ACAN	1:1000	ab36861	Abcam	USA	AB_722655
MMP3	1:1000	YT4465	Immunoway	USA	AB_2935876
ADAMTS5	1:1000	Ab41037	Abcam	USA	AB_2222327
β-actin	1:5000	AC026	Abclonal	USA	AB_2768234

Cleaved Caspase 3	1:500	19677-1-AP	Proteintech	USA	AB_10733244
Cleaved Caspase 7	1:500	27155-1-AP	Proteintech	USA	AB_2880779
NLRP3	1:1000	A12694	Abclonal	USA	AB_2759538
Cleaved Gasdermin D	1:500	36425	CST	USA	AB_2799099
Cleaved Caspase 1	1:500	AF4005	Affinity	USA	AB_2845463
p-MLKL	1:1000	AP0949	Abclonal	USA	AB_2863859
MLKL	1:1000	A5579	Abclonal	USA	AB_2766355
p-RIPK3	1:1000	AF7443	Affinity	USA	AB_2843883
RIPK3	1:1000	DF10141	Affinity	USA	AB_2840721
p65	1:1000	A11204	ABclonal	USA	AB_2758458
p-p65	1:1000	AP0124	ABclonal	USA	AB_2771510
IkBα	1:1000	10268-1-AP	Proteintech	USA	AB_2151423
p-IkBα	1:1000	82349-1-RR	Proteintech	USA	AB_3073626
Histone H3	1:1000	GB11102-100	Servicebio	China	AB_3073627
p-AMPKα1	1:1000	YP0010	Immunoway	USA	AB_3076534
AMPKα1	1:1000	YT0215	Immunoway	USA	AB_3076535

Supplementary Table 4 The primary antibodies for immunohistochemical staining

Name	Dilution	Lot Number	Company	Country	RRID
COL2A1	1:100	YT1022	Immunoway	USA	AB_2935872
ACAN	1:100	A8536	Abclonal	USA	AB_2768197
MMP3	1:100	YT4465	Immunoway	USA	AB_2935876
ADAMTS5	1:100	A2836	Abclonal	USA	AB_2764666
PGC-1α	1:100	66369-1-Ig	Proteintech	USA	AB_2828002
ABAT	1:100	A5299	Abclonal	USA	AB_2766111
UPB1	1:100	A15452	Abclonal	USA	AB_2762360
Cleaved Caspase 3	1:100	19677-1-AP	Proteintech	USA	AB_10733244
Cleaved Gasdermin D	1:100	36425	CST	USA	AB_2799099
p-MLKL	1:100	AP0949	Abclonal	USA	AB_2863859

NLPR3	1:100	A12694	Abclonal	USA	AB_2759538

Supplementary Table 5 The primary antibodies for immunofluorescence

Name	Dilution	Lot Number	Company	Country	RRID
COL2A1	1:100	YT1022	Immunoway	USA	AB_2935872
MMP3	1:100	YT4465	Immunoway	USA	AB_2935876
Cleaved Caspase 3	1:100	19677-1-AP	Proteintech	USA	AB_10733244
Cleaved Gasdermin D	1:100	36425	CST	USA	AB_2799099
p-MLKL	1:100	AP0949	Abclonal	USA	AB_2863859
PGC-1α	1:100	66369-1-Ig	Proteintech	USA	AB_2828002
ABAT	1:100	A5299	Abclonal	USA	AB_2766111
UPB1	1:100	A15452	Abclonal	USA	AB_2762360
